# Emergency Medical Services Clinicians and COVID-19 Booster Behavior—A Cross-Sectional National Evaluation

**DOI:** 10.3390/vaccines13050457

**Published:** 2025-04-25

**Authors:** Gregory Muller, Christopher B. Gage, Jonathan R. Powell, Sarah R. MacEwan, Laura J. Rush, Eben Kenah, Gennaro Di Tosto, Ann Scheck McAlearney, Ashish R. Panchal

**Affiliations:** 1 Section of Prehospital and Disaster Medicine, Department of Emergency Medicine, Stanford University, Stanford, CA 94305, USA; gmuller@stanford.edu; 2 National Registry of Emergency Medical Technicians, Columbus, OH 43229, USA; cgage@nremt.org (C.B.G.); jpowell@nremt.org (J.R.P.); 3 Division of Epidemiology, College of Public Health, The Ohio State University, Columbus, OH 43210, USA; 4 Division of General Internal Medicine, College of Medicine, The Ohio State University, Columbus, OH 43210, USA; sarah.macewan@osumc.edu; 5 The Center for the Advancement of Team Science, Analytics, and Systems Thinking in Health Services and Implementation Science Research (CATALYST), College of Medicine, The Ohio State University, Columbus, OH 43210, USA; laura.rush@osumc.edu (L.J.R.); gennaro.ditosto@osumc.edu (G.D.T.); ann.mcalearney@osumc.edu (A.S.M.); 6 Department of Family and Community Medicine, College of Medicine, The Ohio State University, Columbus, OH 43210, USA; 7 Division of Biostatistics, College of Public Health, The Ohio State University, Columbus, OH 43210, USA; kenah.1@osu.edu; 8 Department of Biomedical Informatics, College of Medicine, The Ohio State University, Columbus, OH 43210, USA; 9 Department of Emergency Medicine, Wexner Medical Center, The Ohio State University, Columbus, OH 43210, USA

**Keywords:** emergency medical services (EMS), COVID-19, workforce readiness

## Abstract

Background/Objectives: Emergency Medical Services (EMS) clinicians in the US have high COVID-19 vaccine hesitancy rates and often do not receive primary vaccinations or subsequent boosters. The extent of booster attrition following initial vaccination and first booster dose in EMS clinicians is unknown. Our objective was to evaluate the prevalence and drivers of COVID-19 booster attrition in EMS clinicians. We hypothesized that booster attrition is common among EMS clinicians and associated with various EMS characteristics. Methods: This study was a cross-sectional analysis of nationally certified civilian EMS clinicians aged 18–85 years old. An electronic survey was distributed, which included an evaluation of vaccination status, booster acceptance, willingness to receive future boosters, perceived risk of contracting COVID-19 from the Understanding America Survey (8 items), and mistrust of healthcare organizations using the Medical Mistrust Index (MMI) (7 items). These data were combined with demographic and work-related characteristics from the National Registry of EMTs dataset. A multivariable logistic regression model (OR, 95% CI) was used to describe booster attrition associations between demographics, work-related characteristics, perceived risk, and medical mistrust. Results: A total of 1902 respondents met initial inclusion criteria. Within this group, 78% were COVID-19 vaccinated, and an additional 65% received a booster. Of these, 37% reported not planning to receive any other booster treatments following the first booster. Primary reasons for not continuing with subsequent boosters include confusion among experts on efficacy (59%), severe side effects (38%), the belief that COVID-19 is not a threat (26%), the belief in natural immunity (25%), and the belief that boosters are not required (23%). Odds of planning to receive another booster increased with receiving a flu vaccine (5.03, 3.08–8.22) and urban environment (1.96, 1.19–3.24, referent rural). In comparison, the odds of planning to receive another booster were lower for paramedics (0.56, 0.38–0.83, referent EMT) and fire agencies (0.53, 0.31–0.89, referent hospital). As the perceived risk of COVID-19 and medical mistrust decrease, the odds of planning to receive another booster increase (perceived risk: 1.98, 1.41–2.78; trust: 4.12, 3.21–5.28). Conclusions: The rate of booster attrition following receipt of one booster is high, at 37%. While there are associations with EMS demographic and workforce characteristics, further exploration is necessary to define the drivers and potential consequences of high booster attrition in the EMS clinician community.

## 1. Introduction

Novel and recurrent infectious diseases represent an ongoing concern to healthcare systems worldwide [1,2]. One common method for mitigating infectious disease is vaccination, a low-cost, widely utilizable resource for controlling pathogen spread [3]. As seen with COVID-19, measles/mumps/rubella (MMR), and other vaccinations, vaccine hesitancy has been increasing on a population level, further presenting challenges to controlling the spread of disease [4,5]. Prior to the availability of COVID-19 vaccines in the United States (US), 23–40% of individuals reported being unsure about receiving the vaccine, and 7–14% reported that they were unlikely or definitely unwilling to receive the COVID-19 vaccine [6,7,8]. A systematic review of COVID-19 vaccine acceptance rates demonstrated large variability around the globe, ranging from 24 to 97% acceptance [9]. Emergency Medical Services (EMS) clinicians, a high-risk population for exposure to recurrent and novel pathogens, faced similar vaccine hesitancy challenges [10,11,12].

Hesitancy among EMS clinicians to receive recurrent and novel vaccinations has been demonstrated to be particularly high [13], with a prior survey of firefighters, emergency medical technicians (EMTs), and paramedics noting that 24% of individuals were unsure about the acceptability of the COVID-19 vaccine, and 27% indicating low acceptability [13]. The initial receipt of the COVID-19 vaccination among nationally registered EMS clinicians demonstrated that only 70% received the vaccine [13]. Other research has demonstrated that the reasons for COVID-19 vaccination hesitancy are multifactorial [10], including concerns regarding vaccine safety, beliefs that the vaccination was not necessary, and associated personal considerations such as one’s own perceived risk and medical mistrust.

After the initial COVID-19 vaccination, two distinct booster doses were released and recommended by the Centers for Disease Control and Prevention (CDC [14]) and the World Health Organization [15]. At the time of the study, an individual compliant with CDC recommendations would have received the initial vaccination series and two booster doses. Internationally, there is significant concern regarding vaccine and booster uptake, with studies demonstrating attrition rates increasing at second and third doses with differences by age and sex [16,17,18]. Regarding healthcare workers, the extent of COVID-19 booster acceptance is less clear, with some maintaining high rates of uptake (>93%) [19], possibly due to mandatory vaccination programs, while others are lower, at approximately 65% [20,21]. However, the evolving nature of vaccine hesitancy and subsequent booster use among EMS clinicians is unknown. We aimed to assess the extent of booster attrition among EMS clinicians after receiving initial COVID-19 vaccination series and first booster dose.

## 2. Materials and Methods

### 2.1. Study Design, Population, and Setting

This study is a cross-sectional evaluation of COVID-19 vaccination booster receipt among a sample of EMS clinicians across the US. We asked questions about initial COVID-19 vaccination and subsequent booster status, demographics, workforce characteristics, and beliefs concerning COVID-19 boosters. We measured beliefs using three validated scales regarding perceived COVID-19 risk, confidence in boosters, and medical mistrust.

The survey was distributed on 5 July 2023, to civilian EMTs and paramedics aged 18–85 in the National Registry of Emergency Medical Technicians (National Registry) database. This distribution occurred after a second booster dose had been officially recommended by the CDC. Participants were included for analysis if they responded to the survey, indicated they had received an initial COVID-19 vaccination, and indicated that they either had or had not received a subsequent booster.

The National Registry provides initial EMS certification to over 95% of U.S. states and territories, and is the primary initial certification agency of EMS clinicians. The National Registry maintains the largest EMS clinician registration database, with over 500,000 registered at the time of the study. Based on previous survey studies conducted, we anticipated low response rates to the survey, and after including an inflation factor (0.08) based on prior work [13], we selected a simple random sample of 21,985 nationally certified EMS clinicians drawn from the database to ensure sufficient respondents.

We sent emails containing a personalized survey link to this random sample of EMS clinicians. No information beyond what was required for disclosure was given to minimize response bias. Follow-up reminder emails were sent following the tailored Dillman methodology. To assess for non-response bias, an abbreviated survey was also administered to all potential respondents who did not respond to the initial or two reminder email invitations. The survey was open for 1 week after the primary survey closure, with all those not responding to initial invitations being solicited for a response. Clinicians’ completion of the primary or non-responder survey had no impact on National EMS Certification. The American Institutes for Research’s Institutional Review Board granted an exemption for this study.

### 2.2. Measures

We determined COVID-19 vaccination and booster status by asking the yes/no binary questions: “Have you received a COVID-19 vaccine?” and “Have you received a COVID-19 booster?” Depending on their responses to these two questions, participants were then asked about their choices regarding receipt of a COVID-19 booster. Participants could mark all that apply on a list of response options. Boosted participants were also asked about the timing of any booster dosing, if they received their booster dose as soon as they were eligible or if they waited. Participants who reported that they waited (or were waiting) were additionally asked why using a mark-all-that-apply format.

We assessed the perceived risk of COVID-19 by adapting a scale of perceived threat and risk of contracting COVID-19 from the Understanding America Survey. Our adapted version asked participants to rate their perceived risk for eight items associated with COVID-19 infection on a four-point response scale (1 = almost no chance, 4 = very high chance). We used the Medical Mistrust Index, a validated seven-item tool, to measure trust in the medical system. We adapted this measure to use a 5-point Likert scale for trust (1 = strongly agree, 5 = strongly disagree).

Demographic data were collected from respondents’ National EMS Certification database profile and linked to the survey results using a common identifier. Variables included sex, age, race/ethnicity, urbanicity (urban vs. rural/suburban), certification level (EMT or paramedic), and education level. Sex was dichotomized as male or female. Age was categorized into quartiles for modeling. In EMS, there is a small number of minority clinicians, therefore race and ethnicity were dichotomized to non-minority (white, non-Hispanic) or minority. The minority category included those who identified as Black or African American, Asian, Hispanic or Latino, Asian, or Native Hawaiian or Pacific Islander. Educational levels were grouped into four categories: less than high school/completed high school/obtained GED, some college education, associate’s degree, and bachelor’s degree or above. Agency types were categorized into fire, governmental non-fire, hospital, private, and other.

### 2.3. Analysis

Survey results were merged with demographic characteristics from the National EMS Certification database, and all identifying data were removed to generate a deidentified analysis dataset. From this deidentified dataset, descriptive statistics were evaluated for demographics, initial vaccine receipt, subsequent booster receipt, and beliefs surrounding boosters. For Likert scales, items were reverse coded, when necessary, with mean composites reported.

Subsequently, using multivariable logistic regression, we assessed for variables associated with COVID-19 booster attrition (dichotomous variable), defined as being unwilling to receive an additional COVID-19 booster following completion of the initial COVID-19 vaccination and first booster. Model selection was undertaken using a literature-informed process including variables previously identified as associated with COVID-19 vaccinations among similar populations and sampling methodologies. Odds ratios (ORs) and 95% confidence intervals (95% CIs) were calculated. Model fit was evaluated using the Hosmer–Lemeshow test and receiver operating characteristic curves with data [22].

To ensure that our estimates reflected the characteristics of the overall population, we also conducted a survey-weighted analysis based on the nationally certified EMS population demographics from the National Registry’s recertification dataset. Survey weights were calculated for the following demographic variables: age, gender, race, education, agency type, and number of EMS jobs. Weights were computed as the ratio of national to survey population proportions of each variable’s subgroups (e.g., education level, agency type). Composite base weights were then assigned to every individual to account for the multiple variables/subgroups in the dataset. A sampling-weighted logistic regression analysis was conducted with the variables from the final model defined above. All analyses were completed using Stata/SE version 18 (Stata Corp LP, College Station, TX, USA).

## 3. Results

**Figure 1:**
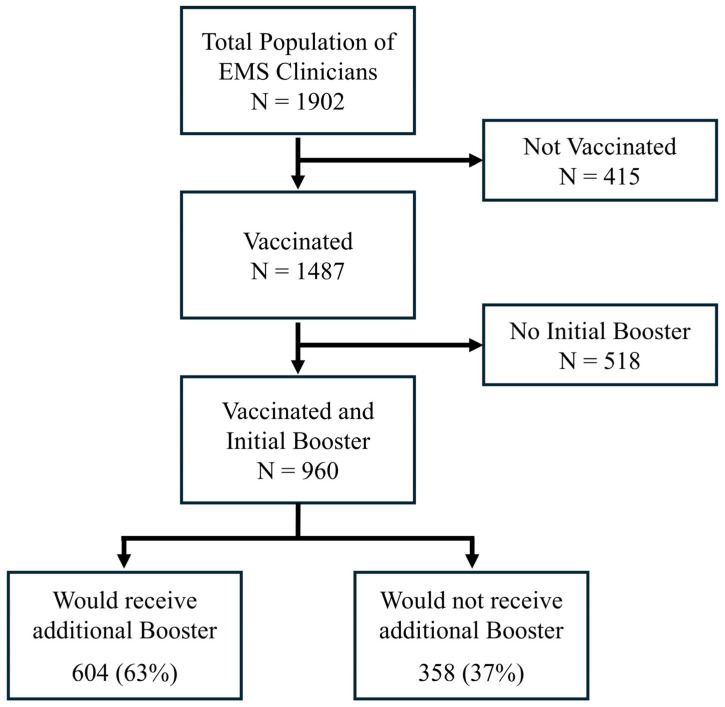
Flowchart of study population. Of the 1902 EMS clinicians surveyed, 960 received the initial COVID-19 vaccination and booster. Of these, 37% would not receive another additional booster.

A total of 1902 participants met the inclusion criteria (response rate = 13%) (Figure 1). Of this population, 78% (n = 1487) received their initial COVID-19 vaccination, and 65% (n = 960) received both initial COVID-19 vaccination and an initial booster.

As shown in Table 1, participants were mostly male (68%, n = 652) and paramedics (67%, n = 637). The population operated mainly in rural/suburban environments (81.8%, n = 781) and predominantly worked for a fire (32%, n = 288) or private (24%, n = 211) agency. Most (86%, n = 804) had received an influenza vaccination, and a minority (38%, n = 363) had at least one comorbidity associated with increased probability of complications of COVID-19.

In terms of beliefs regarding receiving the initial COVID-19 booster vaccination doses, participants reported they most often chose to receive the booster for their protection (68.1%), for the protection of others (56.7%), or pressure from work (35.6%).

### 3.1. Prevalence of COVID-19 Booster Attrition

**Figure 2:**
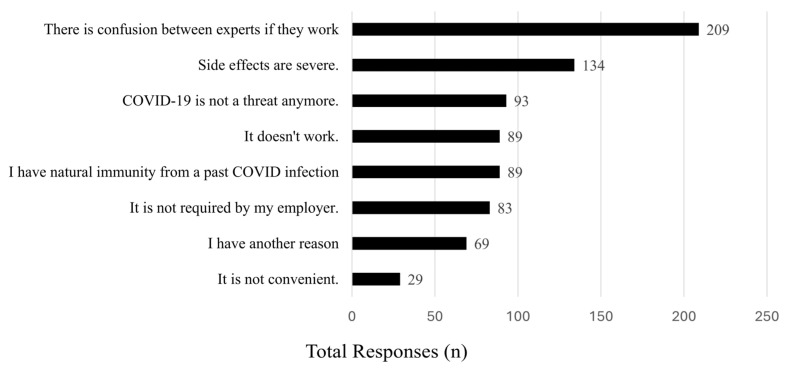
Reasons survey participants, who have already received a COVID-19 vaccine and booster, chose not to receive another additional booster if recommended.

Of the eligible participants who received the COVID-19 vaccination and an initial booster, 37% (n = 358) reported they did not plan to receive an additional COVID-19 booster (Figure 1). The reasons for not receiving a booster dose are summarized in Figure 2.

The most common reasons cited for not continuing to receive booster doses were confusion among experts regarding efficacy (59%, n = 209), severity of side effects (38%, n = 134), the belief that COVID-19 was no longer a threat (26%, n = 93), and belief in natural immunity (25%, n = 89). Participants who reported being unwilling to receive an additional booster dose also reported being highly unlikely (81.6%, n = 292) to be comfortable about getting one under any circumstance.

### 3.2. Predictors of Receiving Additional Boosters

We also evaluated the association between booster attrition and the population’s demographic, personal, and workforce characteristics (Table 2).

Odds of booster attrition, defined as being unwilling to receive an additional COVID-19 booster following completion of the initial vaccination and booster, decreased with receiving an influenza vaccine (OR: 0.20 [95% CI: 0.12–0.33]), urban environment (OR: 0.58 [95% CI: 0.36–0.93], referent rural) and having a bachelor’s degree (OR: 0.45 [95% CI: 0.25–0.81], referent HS/GED or less). Additionally, as the perceived risk of COVID-19 and medical mistrust decreased, the odds of booster attrition decreased (perceived risk: OR: 0.51 [95% CI: 0.37–0.72]; trust: OR: 0.24 [95% CI: 0.19–0.31]). In comparison, the odds of booster attrition increased for paramedics (OR: 1.65 [95% CI: 1.12–2.44], referent EMT) and working in a fire agency (OR: 1.89 [95% CI: 1.12–3.20], referent hospital).

### 3.3. Survey-Weighted Analysis and Non-Responder Survey

Recognizing the challenges of having a nationally representative EMS clinician sample to survey, we mitigated this by conducting a survey-weighted analysis based on the nationally certified EMS population demographics from the National Registry’s recertification dataset (Appendix A.1). Appendix A.2 presents the survey-weighted multivariable logistic regression analysis for booster attrition. Similar to our primary analysis, booster attrition was associated with paramedic certification level and fire agency type. The odds of booster attrition decreased with a bachelor’s degree, an urban environment, and high perceived COVID-19 risk and trust.

We also conducted a non-responder survey to understand the directionality of our sampling bias. The survey was completed by 747 EMS clinicians from the original survey population. Among the non-responders, 396 EMS clinicians received both a vaccine and a booster. Of the participants who received a vaccination and their first booster, 40.7% reported being unwilling to receive a subsequent booster even if recommended. This finding is slightly higher than that of the main survey population, which is 37% (Figure 1).

## 4. Discussion

The public health challenges posed by the COVID-19 pandemic, now evolving into an endemic stage in the US, are complicated by persistent hesitancy toward vaccinations and booster doses. In this study, we conducted a broad survey of EMS clinicians to identify the extent of booster hesitancy among the population, as well as to identify reasons for this hesitancy and determine if any demographic or other factors influenced booster attrition. We found the rate of COVID-19 booster attrition following receipt of the complete initial COVID-19 vaccination series and one booster is high, at 37%. We identified that the main reasons for booster attrition were confusion among experts regarding vaccinations/boosters and booster side effects. Further, we noted increased odds of booster attrition among paramedics and EMS clinicians working in a fire agency. In contrast, as education level, medical trust, and perceived risk of COVID-19 increased, booster attrition decreased. These data are beginning to identify targets for population-based interventions to improve booster use. However, additional work is needed to understand the best method to reach populations with high booster attrition.

This is the first study explicitly addressing booster attrition within the high-risk EMS population that frequently interacts with the public. While vaccinations remain effective, reinfections with COVID-19, including among appropriately vaccinated individuals, are well documented [23]. Our results are similar to several general population studies demonstrating high booster hesitancy in the United States [24]. Further, in international studies of healthcare workers, a pooled analysis of 13 studies that included 12,616 individuals showed that COVID-19 vaccine booster acceptance was 66% [25]. Specific to healthcare workers in the US, a national evaluation demonstrated that only 8% were unwilling to receive the vaccine and subsequent boosters [26]. Our data show a very different picture for EMS clinicians, consistent with our previous evaluation regarding vaccine hesitancy [13]. Based on these results, EMS clinicians will remain particularly vulnerable to novel COVID-19 variants. Inadequate vaccination coverage among this workforce increases the risk of infection, the chance of severe illness, and the likelihood of post-COVID-19 complications with repeated exposures, posing challenges given their critical role in protecting public health.

Our analysis explored why individuals who completed their primary vaccine series and received an initial booster opted not to pursue additional doses. The most common reason for not continuing to receive vaccination booster doses was confusion among experts regarding efficacy (59%). This finding strongly contrasts with available data regarding the effectiveness of the COVID-19 vaccine and overall vaccinations. The cause of this is unclear and may be multifactorial. As noted in this and other work, high medical mistrust may play a significant role [13]. Additionally, low perceived risk was a major theme, with 26% believing that COVID-19 was no longer a significant threat and 25% relying on natural immunity, forming a combined majority view (51%). These findings suggest that many EMS clinicians maintain either a belief in low personal risk or low general threat of COVID-19, which could contribute to low booster uptake.

Addressing these challenges may require targeted communication strategies for populations with low perceived risk. Our findings also indicate that individuals with higher educational attainment were more likely to continue receiving boosters, suggesting that public health efforts might benefit from focused outreach toward groups less inclined to maintain vaccination adherence [11,27]. A recent systematic review and meta-analysis evaluated strategies to increase vaccine uptake [28]. They noted that the most effective strategies include improving access and providing vaccination incentives. Interestingly, common approaches such as reminders, information provision, activation motivation, and misinformation correction were not effective approaches. Tailoring clear, evidence-based messages to address both confusion around efficacy and individual risk could improve vaccination adherence [28], but will need to be built with population acceptability in mind.

### Limitations

Our evaluation has several limitations. First, the data collected in this study are self-reported by EMS clinicians concerning their vaccination status, demographic information, and agency-level characteristics. Reliance on self-reported data may have resulted in misclassification of respondents and social desirability bias. However, the risk of such bias may be mitigated in this study through the anonymous, personally invited survey design.

Second, our sample included only nationally certified EMS clinicians, and since all states do not require this certification, these results may not be fully generalizable. Compared to a national demographic study of EMS clinicians [29], our sample had a higher proportion of individuals with a bachelor’s degree or higher degrees (35% vs. 26%), fewer working in fire departments (32% vs. 48%), and more working in governmental non-fire agencies (16% vs. 12%) and hospitals (14% vs. 11%). These differences, while small, may suggest that national population estimates may have lower vaccination rates than our study population.

Third, our survey response rate was relatively low, a common issue in EMS research. While this response rate is expected [30], it raises the potential for response and selection bias. Still, prior work has suggested these concerns may be unfounded [31], and our analysis of a survey of non-responders demonstrated no difference in our primary outcome of vaccination, supporting the validity of the results we report.

Finally, our survey was conducted at a single time point and captured respondents’ answers and beliefs. Given the ongoing and evolving nature of the COVID-19 pandemic in the US, future research may be warranted to examine changing attitudes about COVID-19 and COVID-19 vaccines over time.

## 5. Conclusions

Infectious diseases like COVID-19 continue to pose a public health challenge, and EMS clinicians face high exposure risk to new COVID-19 variants. We found the booster attrition rate after receiving one booster dose is high, at 37%, and varies based on demographic and workforce characteristics. This suggests that targeted interventions for EMS clinicians may be needed to address booster hesitancy and ensure adequate coverage in this high-risk population. Additionally, the integration of educational or policy-level interventions for EMS agencies may also assist in mitigating booster attrition. Further exploration is necessary to define approaches to address booster attrition in the EMS clinician community.

## Figures and Tables

**Table 1:** Demographics.

	Initial Vaccine and at Least One Booster N = 958 n (%)	Two Boosters Received N = 603 n (%)	Booster Attrition N = 355 n (%)
Age, years (median, IQR)	47 (35, 58)	48 (35, 59)	46 (36, 55)
Male	652 (68.3)	399 (66.5)	253 (71.5)
Certification level: paramedic	637 (66.5)	381 (59.8)	256 (40.2)
Education level			
HS/GED or less	89 (9.7)	44 (7.6)	45 (13.2)
Some college education	244 (26.5)	148 (25.6)	96 (28.1)
Associate’s	190 (20.6)	103 (17.8)	87 (25.4)
Bachelor’s or above	398 (41.5)	284 (47.1)	114 (32.1)
Urban			
Rural/suburban	781 (81.8)	480 (79.9)	301 (85.0)
Urban	174 (18.2)	121 (20.3)	53 (15.0)
Agency			
Hospital	137 (15.3)	97 (17.2)	40 (12.1)
Fire	288 (32.2)	156 (27.6)	132 (40.0)
Government non-fire	143 (16.0)	94 (16.6)	49 (14.9)
Private	211 (23.6)	141 (25.0)	70 (21.2)
Other	116 (13.0)	77 (13.6)	39 (11.8)
Flu vaccine received	804 (85.5)	550 (93.5)	254 (72.2)
Presence of COVID-19 comorbidity	363 (38.1)	242 (40.3)	121 (34.4)

**Table 2:** Odds ratios and 95% confidence intervals (CI) for booster attrition. Odds ratios less than 1 indicate reduced likelihood of booster attrition.

	OR (95% CI)	*p* Value
Certification (paramedic)	1.65 (1.12–2.44)	*0.010* *
Education level		
HS/GED or less	Referent	
Some college education	0.59 (0.32–1.09)	0.091
Associate’s	0.84 (0.45–1.57)	0.596
Bachelor’s or above	0.45 (0.25–0.81)	*0.007* *
Urbanicity		
Rural/suburban	Referent	
Urban	0.58 (0.36–0.93)	*0.023* *
Agency type		
Hospital	Referent	
Fire	1.89 (1.12–3.20)	*0.018* *
Government non-fire	1.44 (0.79–2.66)	0.237
Private	1.01 (0.57–1.78)	0.969
Other	1.14 (0.59–2.20)	0.699
Flu vaccine received	0.20 (0.12–0.33)	*<0.001* *
Mean risk	0.51 (0.37–0.72)	*<0.001* *
Mean trust	0.24 (0.19–0.31)	*<0.001* *

Abbreviations: GED—general educational development, HS—high school. Goodness-of-fit test: *p* = 0.4161; likelihood ratio test of area under the receiver operating characteristic curves: 0.8269. *, *p* < 0.05.

## Data Availability

The data presented in this study are only available on request from the corresponding author due to data sharing policies through the National Registry of EMTs.

## Abbreviations

The following abbreviations are used in this manuscript:

CDC	Centers for Disease Control and Prevention
CI	Confidence interval
COVID-19	Coronavirus disease 2019
EMS	Emergency Medical Services
EMT	Emergency Medical Technician
GED	General educational development
HS	High school
IQR	Interquartile Range
MMI	Medical Mistrust Index
MMR	Measles, mumps, and rubella
OR	Odds ratio
US	United States

## Appendix A

### Appendix A.1

**Table A1:** Booster attrition and total recertifying populations used in survey-weighting analysis.

Variables	Booster Attrition Population N = 958	Total Recertifying Population N = 116,682
Age, years (median, IQR)	47 (35, 58)	35 (28, 45)
Male	652 (68.3)	83,766 (72.1)
Education level		
HS/GED or less	89 (9.7)	18,262 (15.7)
Some college education	244 (26.5)	38,231 (32.8)
Associate’s	190 (20.6)	23,478 (20.1)
Bachelor’s or above	389 (43.2)	36,613 (31.4)
Urban		
Rural/suburban	781 (81.8)	104,717 (89.7)
Urban	174 (18.2)	11,965 (10.3)
Agency		
Hospital	137 (15.3)	12,167 (11.4)
Fire	288 (32.2)	51,589 (48.5)
Government non-fire	143 (16.0)	12,586 (11.8)
Private	211 (23.6)	21,114 (19.9)
Other	116 (13.0)	8868 (8.3)

### Appendix A.2

**Table A2:** Survey-weighted sample odds ratios and 95% confidence intervals (CI) for booster attrition. Odds ratios less than 1 indicate reduced likelihood of booster attrition.

	OR (95% CI)	*p* Value
Certification (paramedic)	1.89 (1.13–3.15)	*0.015*
Education level		
HS/GED or less	Referent	
Some college education	0.49 (0.22–1.08)	0.078
Associate’s	0.56 (0.25–1.30)	0.178
Bachelor’s or above	0.46 (0.22–0.96)	*0.037*
Flu vaccine received	0.16 (0.09–0.29)	<0.001
Urban	0.54 (0.30–0.96)	*0.036*
Agency type		
Hospital	Referent	
Fire	2.13 (1.04–4.34)	*0.038*
Government non-fire	1.50 (0.68–3.34)	0.315
Private	0.84 (0.38–1.84)	0.658
Other	1.72 (0.76–3.89)	0.195
Mean risk	0.61 (0.40–0.95)	*0.029*
Mean trust	0.25 (0.17–0.35)	*<0.001*
